# In vivo monitoring of dynamic interaction between neutrophil and human umbilical cord blood-derived mesenchymal stem cell in mouse liver during sepsis

**DOI:** 10.1186/s13287-020-1559-4

**Published:** 2020-02-03

**Authors:** Sung Yong Ahn, Yong-Sun Maeng, Yu Rim Kim, Young Ho Choe, Han Sung Hwang, Young-Min Hyun

**Affiliations:** 1 Department of Anatomy, Yonsei University College of Medicine, Seoul, Republic of Korea; 2 Department of Obstetrics and Gynecology, Yonsei University College of Medicine, Seoul, Republic of Korea; 3 Institute of Women’s Life Medical Science, Yonsei University College of Medicine, Seoul, Republic of Korea; 4 BK21 PLUS Project for Medical Science, Yonsei University College of Medicine, Seoul, Republic of Korea; 5 Department of Obstetrics and Gynecology, Research Institute of Medical Science, Konkuk University School of Medicine, Seoul, Republic of Korea

**Keywords:** Two-photon intravital imaging, Sepsis, Human umbilical cord blood-mesenchymal stem cells, Neutrophils, Hepatic stellate cells

## Abstract

**Background:**

Sepsis is a global inflammatory disease that causes death. It has been reported that mesenchymal stem cell (MSC) treatment can attenuate inflammatory and septic symptoms. In this study, we investigated how interactions between neutrophils and human umbilical cord blood (hUCB)-MSCs in the liver of septic mice are involved in mitigating sepsis that is mediated by MSCs. Accordingly, we aimed to determine whether hUCB-MSC application could be an appropriate treatment for sepsis.

**Methods:**

To induce septic condition, lipopolysaccharide (LPS) was intraperitoneally (i.p.) injected into mice 24 h after the intravenous (i.v.) injection of saline or hUCB-MSCs. To determine the effect of hUCB-MSCs on the immune response during sepsis, histologic analysis, immunoassays, and two-photon intravital imaging were performed 6 h post-LPS injection. For the survival study, mice were monitored for 6 days after LPS injection.

**Results:**

The injection (i.v.) of hUCB-MSCs alleviated the severity of LPS-induced sepsis by increasing IL-10 levels (*p* < 0.001) and decreasing mortality (*p* < 0.05) in septic mice. In addition, this significantly reduced the recruitment of neutrophils (*p* < 0.001) to the liver. In hUCB-MSC-treated condition, we also observed several distinct patterns of dynamic interactions between neutrophils and hUCB-MSCs in the inflamed mouse liver, as well as vigorous interactions between hepatic stellate cells (HSCs or ito cells) and hUCB-MSCs. Interestingly, hUCB-MSCs that originated from humans were not recognized as foreign in the mouse body and consequently did not cause graft rejection.

**Conclusions:**

These distinct interaction patterns between innate immune cells and hUCB-MSCs demonstrated that hUCB-MSCs have beneficial effects against LPS-induced sepsis through associations with neutrophils. In addition, the immunomodulatory properties of hUCB-MSCs might enable immune evasion in the host. Taken together, our results suggest the prospects of hUCB-MSCs as a therapeutic tool to inhibit inflammation and alleviate pathological immune responses such as sepsis.

## Backgrounds

Sepsis, the result of systemic inflammatory responses induced by infection, which can lead to the clearance of bacteria or pathogens, is a leading cause of death worldwide [1, 2]. The associated exaggerative immune response and pro-inflammatory cytokine overexpression cause tissue damage and lead to various organ dysfunctions [3]. Neutrophil activity and their recruitment to essential organs such as the lung and liver are crucial for the immunopathogenesis of severe sepsis [3, 4]. A gram-negative bacteria-induced sepsis model can be simplified to induction by lipopolysaccharide (LPS) treatment, wherein the recruitment of neutrophils to the liver results in detrimental effects including multi-organ failure and dysfunction [4, 5].

Mesenchymal stem cells (MSCs) are multipotent stromal cells that have important properties including anti-apoptotic, angiogenic, growth factor-inducing, anti-fibrotic, and chemo-attractive activities [6]. MSCs also have immunomodulatory properties [7, 8], and the beneficial effects of human MSCs on septic mice have been demonstrated [9, 10]. However, the immunomodulatory properties of these cells have not been fully elucidated.

Our previous study showed that cord blood-derived mononuclear cells (MNCs) can differentiate into MSCs or outgrowth endothelial cells (OECs) [11]. We also characterized the differentiation potential of CD133^+^/C-kit^+^/Lin^−^ MNCs (CKL^−^ cells) isolated from human umbilical cord blood (UCB) and confirmed that CKL^−^ cells spontaneously differentiate into MSCs or OECs by performing RT-PCR and immunofluorescence staining for the respective specific markers [11]. Based on the reported beneficial effects of human MSCs against sepsis [9, 10], we hypothesized that human umbilical cord blood-derived mesenchymal stem cells (hUCB-MSCs) could alleviate sepsis-associated acute organ and systemic inflammation via their immunomodulatory properties to improve survival in LPS-induced sepsis. Next, we assumed that latent communication mechanisms such as migration, recruitment, and dissociation between innate immune cells and hUCB-MSCs might be required to maintain the immune system balance during sepsis. For this reason, we monitored dynamic interactions between innate immune cells such as neutrophils and hUCB-MSCs in the mouse liver using two-photon intravital imaging. Interestingly, neutrophils can acquire the capacity to function as antigen-presenting cells (APCs) under inflammatory conditions or during association with other cells [12]. In addition, neutrophils have been reported to express the inherent receptors for antigen presentation [12]. Therefore, we speculated that the efficacy of hUCB-MSCs in resolving acute inflammation might be due to immunomodulation through dynamic interactions with neutrophils. Hence, we aimed to assess the various biological behaviors of neutrophils and hUCB-MSCs in a murine sepsis model in real time.

## Methods

### Mice

Female C57BL/6 mice (Orient Bio, Seongnam, South Korea) were used for this study. In a preliminary test, we used both male and female mice; there was no gender difference in the results of the experiment. LysM-GFP [13] mice and CX3CR1-GFP mice [14] were obtained. All mice were maintained under specific-pathogen-free conditions in the animal establishment at Avison Biomedical Research Center in Yonsei University College of Medicine, and all experiments were approved by the Institutional Animal Care and Use Committee of Yonsei University Health System (IACUC 2017-0353).

### Characterization of hUCB-MSCs

hUCB-MSCs were prepared as described previously [11]. Briefly, CKL^−^ cells were purified by positive and negative selection with anti-CD133/C-kit/Lin^−^ microbeads (Miltenyi Biotec, Bergisch-Gladbach, Germany) using a magnetic cell sorter device (Miltenyi Biotec) from cord blood samples. CKL^−^ cells were seeded in 6-well plates coated with human fibronectin (Sigma, St. Louis, MO) in endothelial basal medium-2 (Clonetics, Cell Systems, St. Katharinen, Germany). The medium was supplemented with endothelial growth medium-2 (EGM-2; Clonetics, Cell Systems) containing fetal bovine serum, human VEGF-A, human fibroblast growth factor-B, human epidermal growth factor, IGF1, and ascorbic acid. MSCs differentiated from CKL^−^ cells were identified via staining with PE-CD73, FITC-CD90, and alpha-smooth muscle actin (훼-SMA) (BD Biosciences, Bedford, MA). MSCs (at 5 × 10^6^ cells/dish) were cultivated in 100-mm cell culture dishes coated with 0.01 mg/mL bovine plasma-derived fibronectin (R&D systems, Minneapolis, MN, USA) in endothelial basal medium-2 (EBM-2; Lonza, Basel, Switzerland). MSCs in all studies were used at passage < 10. The sampling and use of medical records for research purpose were performed with the consent of all patients. This study was approved by the Yonsei University Hospital Review Board (4-2005-0186).

### MSC administration

Before (24 h) LPS injection, saline or MSCs (at 2 × 10^6^ cells/200 μL of saline) were slowly infused via the tail vein of mice. Mice were randomly assigned to one of three experimental conditions as follows: (1) saline + saline (control), (2) saline + LPS (LPS-only-treated condition), (3) hUCB-MSCs + LPS (hUCB-MSC-treated condition). Finally, for two-photon intravital imaging experiments, an additional condition in which only hUCB-MSCs were administered was included.

### LPS-induced sepsis

LPS from *Salmonella enterica* serotype enteritidis was purified by phenol extraction (Sigma). To induce sepsis, mice were intraperitoneally injected with LPS at 0.5 mg/kg 24 h after the i.v. injection of saline or hUCB-MSCs. Mice were sacrificed 6 h after LPS injection to acquire tissues and blood for analysis. The lung and liver were extracted and fixed in 3.7% paraformaldehyde for 24 h. Whole blood was acquired from mice by cardiac puncture. To obtain plasma, within 30 min, samples were spun down and the supernatant was transferred and stored at − 80 °C.

### Histologic analysis

After fixation, tissues were embedded in paraffin and sectioned at 4-μm thickness. Hematoxylin and eosin (H&E) staining was conducted to determine inflammatory morphologic changes and to assess the infiltration of inflammatory cells in the lung and liver after acute LPS-induced sepsis. The number of inflammatory cells was counted in nine randomly chosen histological fields per section at an original magnification of × 400.

### Cytokine and chemokine immunoassay

Whole blood was acquired by cardiac puncture and centrifuged at 2000×*g* for 20 min at 4 °C. We conducted an additional centrifugation at 10,000×*g* for 10 min at 4 °C for complete platelet removal. Plasma samples were harvested using a standard procedure. Plasma separation tubes (BD, Franklin Lakes, NJ, USA) with lithium heparin and gel were used for plasma collection. Heparin was used as an anticoagulant. Plasma samples were stored at − 80 °C for analysis. The plasma samples were analyzed for IL-1β, IL-6, IL-8, IL-10, TNF-α, IFN-γ, and CXCL1 via enzyme-linked immunosorbent assay (ELISA) using mouse-specific kits (R&D systems, Minneapolis, MN, USA; MyBioSource, San Diego, CA, USA; Abcam, Cambridge, UK; and AbFRONTIER, Seoul, Republic of Korea).

### Survival study

Female C57BL/6 mice (8–14 weeks old) were slowly infused with saline or hUCB-MSCs (at 2 × 10^6^ cells/200 μL of saline) via i.v. injection. To induce sepsis, mice were i.p. injected LPS at 25 mg/kg 24 h later. Mice were then monitored for 6 days.

### Fluorescent labeling of MSCs

MSCs were stained with red fluorescence using CellTracker CMTPX (Thermo, Waltham, MA, USA). Briefly, harvested and resuspended cells were gently mixed with pre-warmed CellTracker CMTPX (at 1 μM/2 × 10^6^ cells). Then, cells were incubated for 20 min at 37 °C.

### Two-photon intravital imaging of mouse liver

We previously described a staging system and two-photon microscopy to obtain imaging data from the live mouse liver [15, 16]. Two-photon microscopy and Zen software (Carl-Zeiss, Oberkochen, Germany) were used for mouse imaging with an imaging chamber. Fluorescence protein-expressing mice (LysM-GFP and CX3CR1-GFP mice) were administered saline or red-labeled hUCB-MSCs (MSCs-CMTPX) 24 h before LPS i.p*.* injection. Then, LPS (0.5 mg/kg) was injected to induce sepsis 6 h before imaging experiments. Mice were anesthetized using Zoletil at a 30 mg/kg via i.p. injection for imaging. Consequently, two-photon intravital imaging was performed 30 h after the mice were injected with saline or hUCB-MSCs. The mouse liver was imaged for approximately 40–50 min at a wavelength 880–900 nm. The images were obtained at a resolution of 512 × 512 pixels using an interval of 1 μm at a depth of 40 μm every 60 s.

### Imaging data analysis

Analysis of imaging data was conducted using Volocity (PerkinElmer, Waltham, MA, USA) and Fiji/ImageJ software.

### Statistical analysis

Statistical analyses of data were conducted using Prism 8 software (GraphPad). Quantitative data are expressed as means and S.D. of at least three independent experiments. The statistical significance of differences among conditions was determined by a one-way ANOVA with Dunnett’s post hoc test (for histologic analysis and cytokine and chemokine immunoassays). Survival curves were analyzed by a log-rank test. Quantitative analyses of MSCs-CMTPX with LysM-GFP cells were performed by a one-way ANOVA with Dunnett’s post hoc test or a Mann–Whitney test for two-photon intravital imaging.

## Results

### hUCB-MSCs alleviate LPS-induced sepsis

Due to their plasticity and migratory and non-immunogenic properties, hUCB-MSCs are potentially useful for transplantation and the treatment of many diseases [17, 18]. To use these cells in a mouse model of disease, we first cultured hUCB-MSCs and analyzed their characteristics. hUCB-MSCs showed a fibroblastic-like morphology and more spindle-shaped morphology at higher cell density (Additional file 1: Figure S1a). The spindle-shaped appearance of hUCB-MSCs is the result of vimentin expression indicating extracellular matrix synthesis and tissue regeneration [6]. Moreover, hUCB-MSCs expressed the lineage-specific markers CD73, CD90, and α-SMA (Additional file 2: Figure S1b). The expression of these molecules implies the hypo-immunogenicity of hUCB-MSCs, which forms an inhibitory milieu surrounding hUCB-MSCs [19].

**Fig. 1:**
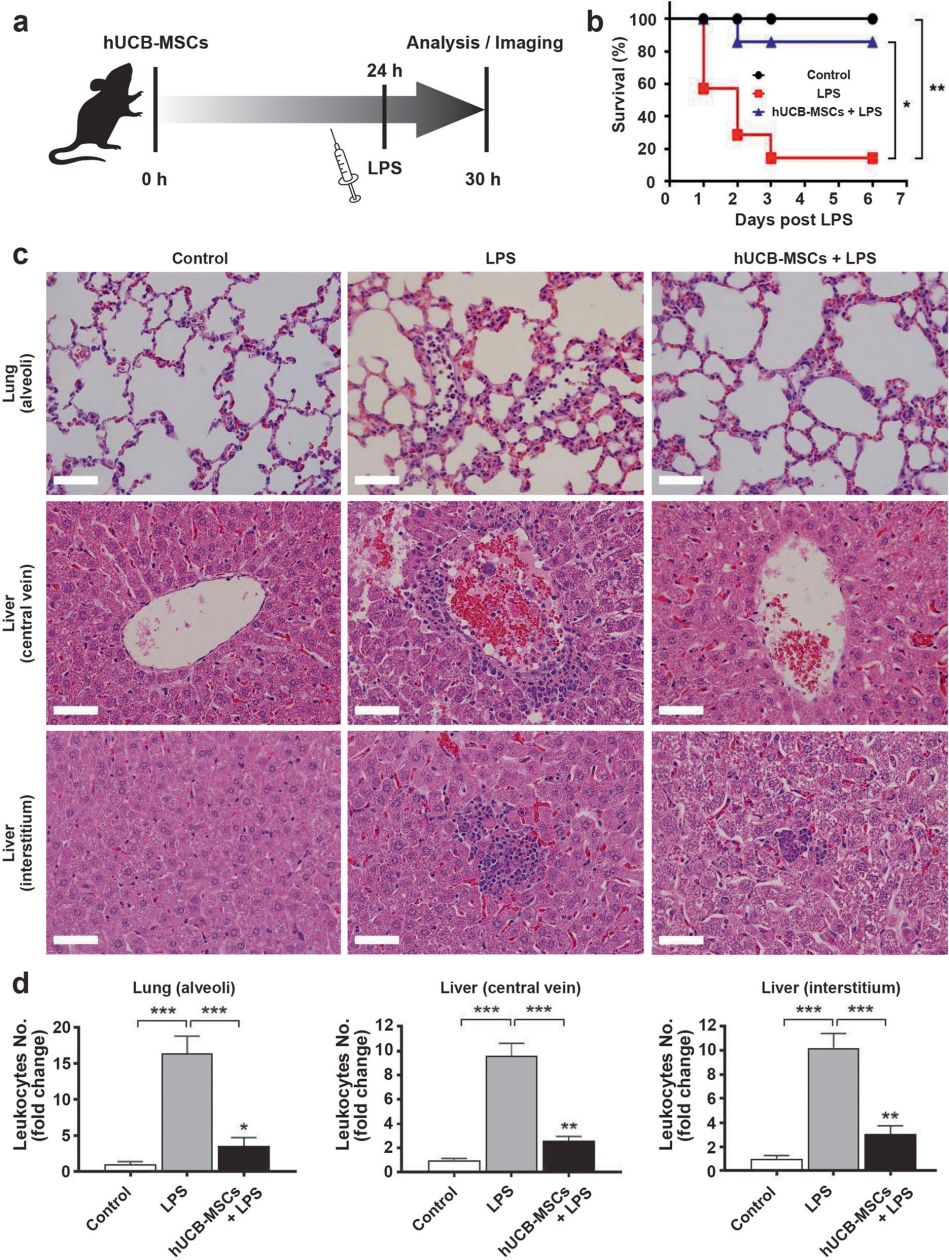
Effects of hUCB-MSC treatment on LPS-induced sepsis. **a** Schematic diagram of the study. Saline or a suspension of hUCB-MSCs was slowly infused into tail veins. Mice with sepsis were intraperitoneally induced with 0.5 or 25 (for survival study) mg/kg of LPS 24 h after hUCB-MSC administration, and 6 h later, they were sacrificed for in vivo experiments or were visualized by two-photon intravital imaging. **b** hUCB-MSC treatment significantly improved survival in the hUCB-MSC-treated condition compared to that in the LPS-only condition. hUCB-MSCs were administered 24 h prior to LPS (25 mg/kg) treatment. Mice were monitored for 6 days; *n* = 7 for each condition. Kaplan–Meier curves were analyzed by a log-rank test. *p < 0.05, **p < 0.005. **c** Pathological inflammatory changes in lung (alveoli) and liver (central vein and interstitium) tissues were shown by H&E staining (original magnification, × 400; scale bar = 50 μm). **d** The graphs show the number of leukocytes for each condition relative to that in control lung (alveoli) and liver (central vein and interstitium) tissues. Quantitative results indicate the average values ± SD of at least three independent experiments. The results were analyzed by a one-way ANOVA with Dunnett’s post hoc test. *p < 0.01 and **p < 0.005 versus control; ***p < 0.001 versus each condition

Multi-organ dysfunction is induced by the massive recruitment of neutrophils during sepsis and severe systemic inflammatory response syndrome [20]. To generate an animal sepsis model, hUCB-MSCs were administered to mice through i.v. injection 24 h before the induction of septic conditions through LPS injection (Fig. 1a). Recently, MSCs have been reported to be efficacious for the treatment of various immune cell function-related diseases including sepsis [21, 22]. To evaluate the effect of hUCB-MSC treatment on survival in LPS-induced septic mice, cells were administered 24 h before LPS injection (25 mg/kg) (Fig. 1b). Survival was significantly improved in the hUCB-MSC-treated condition compared to that in the LPS-only condition, and there was no significant difference between hUCB-MSC-treated and control conditions. The 6-day survival rate of LPS-induced septic mice treated with hUCB-MSCs was 85.7%, which was significantly higher than 14.3% for the LPS-only condition. Thus, hUCB-MSC treatment improved the survival of LPS-induced septic mice.

To determine how hUCB-MSC treatment affects LPS-induced sepsis, histologic analysis of leukocyte infiltration was conducted on lung and liver tissues, which were collected 6 h after LPS injection, and interestingly, sepsis was attenuated (Fig. 1c). According to a recent study, 6 h post-LPS injection, treatment with ASCs (adipose-derived mesenchymal stem cells) and educated macrophages significantly alleviated the levels of LPS-induced pro-inflammatory cytokine IFN-γ and IL-6 while increasing the levels of anti-inflammatory cytokine IL-10 in the serum. This phenomenon peaked at 6 h post-LPS injection and mostly decreased by 48 h [23]. Thus, 6 h was appropriate as the timeline of administration to determine the effect of MSC in inflammation. Acute organ inflammation in the LPS-induced septic mice was determined by counting leukocytes from H&E-stained lung and liver tissue sections. The number of leukocytes in the LPS-only condition was markedly increased in alveoli of the lung, central vein, and interstitium of the liver, when compared to that in controls (Fig. 1d). However, hUCB-MSC treatment significantly decreased these numbers (Fig. 1d). These data imply that hUCB-MSC treatment alleviates LPS-induced sepsis.

**Fig. 2:**
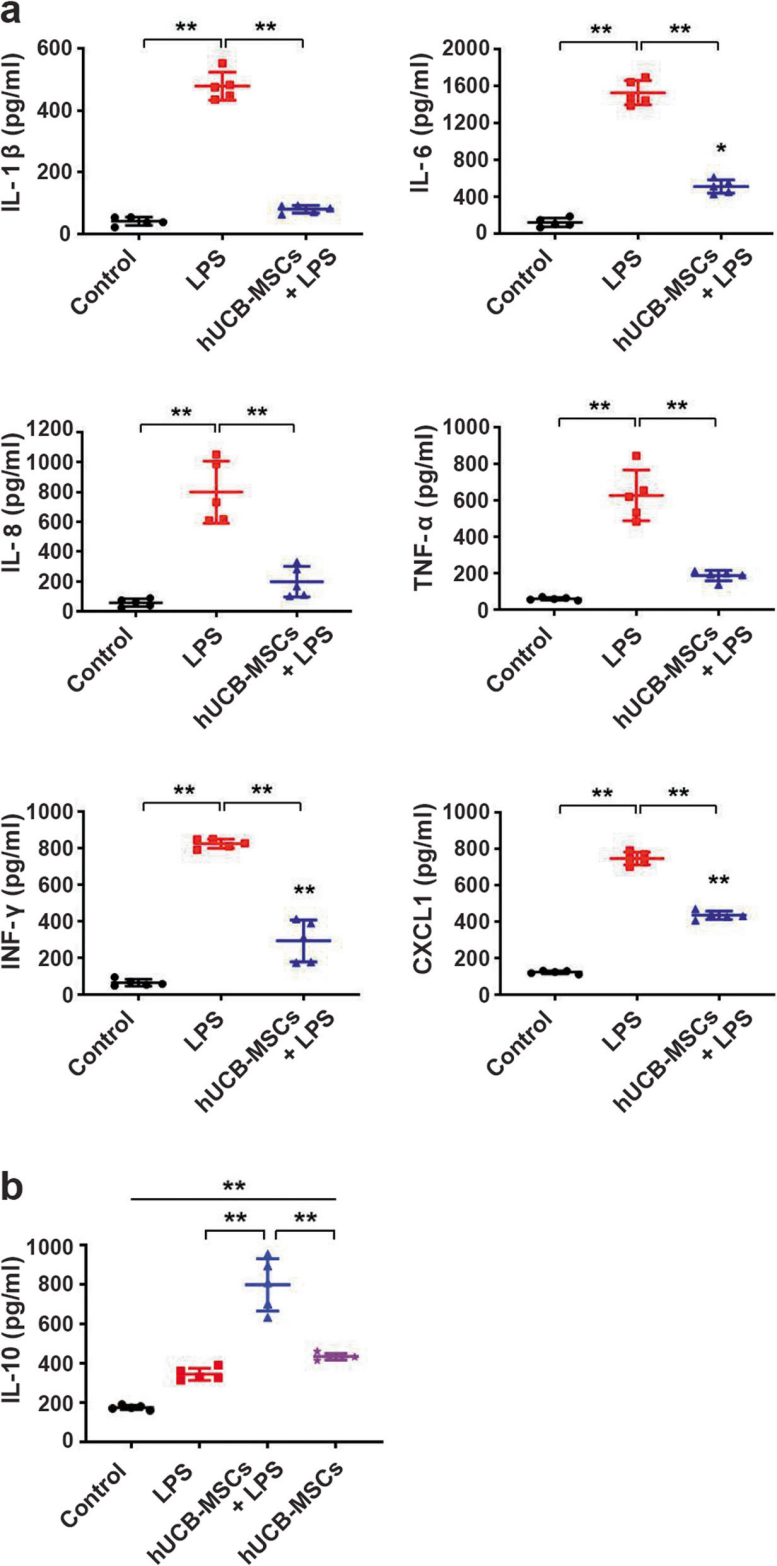
Effects of hUCB-MSC treatment on LPS-induced systemic sepsis and survival. **a** The levels of the pro-inflammatory cytokines and a chemokine (IL-1β, IL-6, IL-8, TNF-α, IFN-γ, and CXCL1) in the plasma. **b** Levels of the anti-inflammatory cytokine IL-10 in the plasma were measured by immunoassay. Quantitative results indicate the average values ± SD of at least three independent experiments. The results were analyzed by a one-way ANOVA with Dunnett’s post hoc test. *p < 0.005, **p < 0.001

To further investigate the effect of hUCB-MSCs on LPS-induced septic conditions, plasma cytokines and chemokines were assayed 6 h post-LPS injection. Compared to those in controls, levels of pro-inflammatory cytokines such as IL-1β, IL-6, IL-8, TNF-α, and IFN-γ were markedly increased with LPS (Fig. 2a). Levels of the pro-inflammatory chemokine CXCL1 were also markedly increased (Fig. 2a). In contrast, hUCB-MSC treatment significantly reduced LPS-induced systemic cytokine and chemokine (IL-1β, IL-6, IL-8, TNF-α, IFN-γ, and CXCL1) levels (Fig. 2a). Interestingly, levels of the anti-inflammatory cytokine IL-10 were increased with LPS. Moreover, treatment with hUCB-MSCs markedly elevated IL-10 levels compared to those in the LPS-treated condition (Fig. 2b). Compared to those in control, the IL-10 levels significantly increased in the condition transferred by hUCB-MSC alone, and there was no significant difference with the LPS-treated condition (Fig. 2b). Therefore, hUCB-MSCs seem to have immunomodulatory properties through IL-10 expression. Together, our findings verified that hUCB-MSC treatment mitigates both pro-inflammatory responses and mortality associated with sepsis.

### hUCB-MSCs attenuate the severity of sepsis-related acute liver injury

The liver is an essential target organ of inflammatory pathology associated with neutrophils during sepsis and systemic inflammation [4, 24, 25]. The hepatic sinusoids that make up most blood vessels in the liver are discontinuous capillaries and are larger in diameter and more irregular in shape than other types of capillaries. Hence, the directional migration of neutrophils in hepatic sinusoids is less restricted than that in other organs and occurs faster. Therefore, the liver is suitable for monitoring the migration patterns of innate immune cells such as neutrophils in inflammatory diseases including sepsis. Neutrophils are recruited to the sinusoidal capillary of the liver during inflammation [26, 27]. To investigate the effect of hUCB-MSCs on neutrophil recruitment under septic conditions, we performed two-photon intravital imaging. It has been reported that low concentrations of LPS (0.5 mg/kg) induce the significant recruitment of leukocytes into the liver without leukocyte-mediated severe tissue damage and high mortality [4, 24]. In addition, our preliminary experiments demonstrated that the number of neutrophils per field of view (FOV) (mm^3^) showed no significant difference between the two different doses (0.5 mg/kg and 25 mg/kg) of hUCB-MSC-treated condition in 24 h post-LPS injection (Additional file 2: Figure S2a and b; Additional file 18: Video S15 and Additional file 19: S16). For that reason, the dose of 0.5 mg/kg was proper to observe the interaction between hUCB-MSCs and neutrophils during sepsis.

**Fig. 3:**
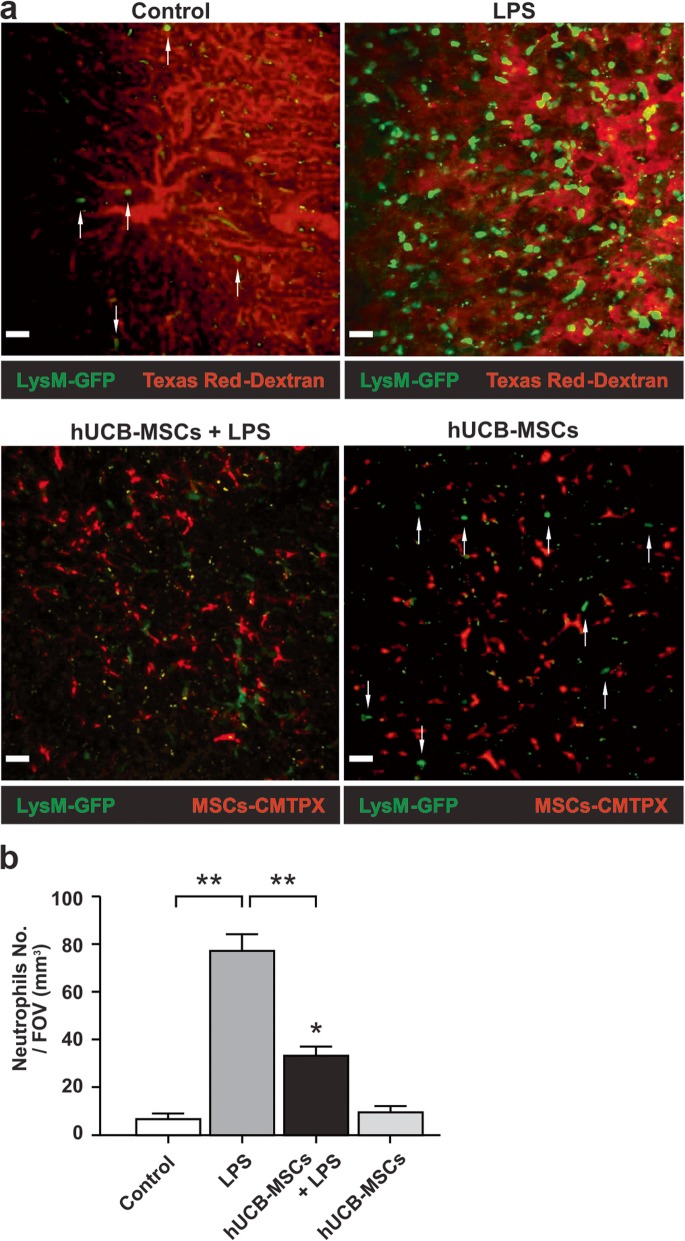
Two-photon intravital microscopy of neutrophils and hUCB-MSC interactions in the liver of LysM-GFP^+/−^ mice. **a** Representative images for each condition; (upper panel) red: hepatic microvasculature (Texas Red-Dextran signal); green: neutrophils (endogenous signal), (lower panel) red: CMTPX-labeled hUCB-MSCs (exogenous signal); green: neutrophils (endogenous signal). Conditions included control (Additional file 4: Video S1), LPS (LPS-only-treated; Additional file 5: Video S2), hUCB-MSCs + LPS (hUCB-MSCs-treated; Additional file 6: Video S3), and hUCB-MSCs (hUCB-MSCs only treated; Additional file 7: Video S4). The “guiding arrows” represent neutrophils in control and hUCB-MSC conditions. These data are representative of three independent experiments (original magnification, × 200; scale bar = 30 μm). **b** The graph shows the number of neutrophils per FOV (mm^3^) for each condition in **a**. Quantitative results indicate the average values ± SD of at least three independent experiments. The results were analyzed by a one-way ANOVA with Dunnett’s post hoc test. *p < 0.01 versus control, **p < 0.001 versus each condition

Based on two-photon intravital imaging of the liver of LysM-GFP^+/−^ mice in a time-lapse manner, neutrophils were mostly observed in the liver bloodstream, but only a small number of neutrophils were observed in the absence of LPS treatment (Fig. 3a, upper left panel; Additional file 4: Video S1). In contrast, LPS-induced sepsis significantly promoted the recruitment of many neutrophils to the liver (Fig. 3a, upper right panel; Additional file 5: Video S2). The number of neutrophils per field of view (FOV) (mm^3^) was markedly higher in the LPS-treated condition than in control (Fig. 3b). This indicates that LPS-induced sepsis promotes neutrophil recruitment to the liver. Using two-photon intravital imaging of the liver in LPS-induced septic LysM-GFP^+/−^ mice, we observed that the injection of hUCB-MSCs significantly reduced neutrophil recruitment compared to that in LPS-only-treated mice (Fig. 3a, lower left panels; Additional file 5: Video S2 and Additional file 6: S3). The number of neutrophils per FOV (mm^3^) was markedly lower in the hUCB-MSC-treated condition than in the LPS-treated condition (Fig. 3b). These observations suggest that fewer neutrophils are recruited to the liver upon hUCB-MSC treatment.

Neutrophils are mainly recruited by damage-associated molecular patterns (DAMPs; sterile attack) or pathogen-associated molecular patterns (PAMPs; microbial attack) to inflamed areas [28]. PAMPs cause neutrophil recruitment through microbial-induced inflammatory responses [29]. Therefore, we added a hUCB-MSC-only condition to confirm that neutrophils recognize hUCB-MSCs as PAMPs. Interestingly, we found almost no difference in the hUCB-MSC-only condition compared to that in control (Fig. 3a, lower right panel; Additional file 7: Video S4). There was no significant difference in the number of neutrophils per FOV (mm^3^) between the hUCB-MSC-only and control conditions (Fig. 3b). This finding strongly implies that neutrophils do not recognize hUCB-MSCs as pathogens.

### hUCB-MSCs exert beneficial effects on LPS-induced sepsis through various migratory behaviors with neutrophils

**Fig. 4:**
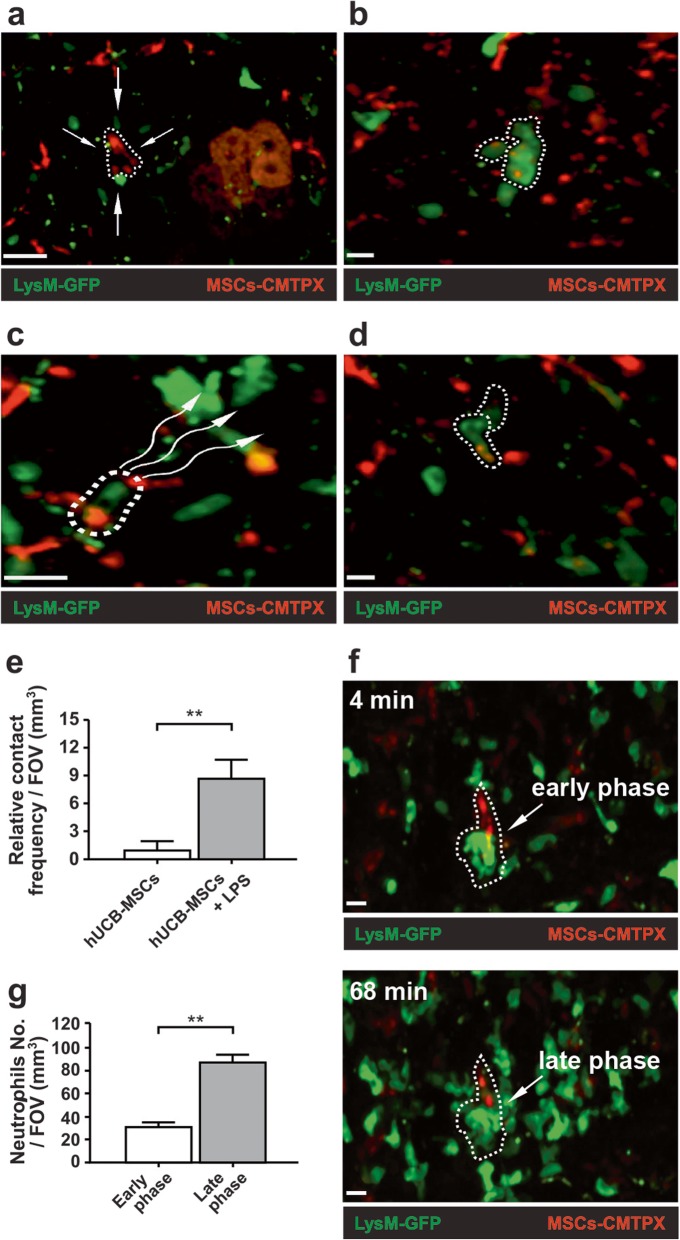
Dynamic interactions between neutrophils and hUCB-MSCs in the inflamed liver of LysM-GFP^+/−^ mice using two-photon intravital microscopy. Representative images of dynamic interactions; red: CMTPX-labeled hUCB-MSCs (exogenous signal); green: neutrophils (endogenous signal). **a** Neutrophils migrated toward the activated hUCB-MSCs upon LPS stimulation (Additional file 8: Video S5). **b** Neutrophils gathering toward hUCB-MSCs attempted to phagocytize hUCB-MSCs (Additional file 9: Video S6 and Additional file 10: S7). **c** Neutrophils that engulfed some hUCB-MSCs migrated from the original site to another site (Additional file 11: Video S8). **d** Neutrophils showed a significant increase in contact frequency with other adjacent neutrophils (Additional file 12: Video S9). **e** The graph shows the relative contact frequency between neutrophils per FOV (mm^3^) in the hUCB-MSC-treated condition (hUCB-MSC only versus hUCB-MSC + LPS). **f** The early phase (4 min) and late phase (68 min) of neutrophil swarming (Additional file 13: Video S10 and Additional file 14: S11). **g** The graph shows the number of neutrophils per FOV (mm^3^) during neutrophil swarming (early phase versus late phase). Quantitative results indicate the average values ± SD of at least three independent experiments. The results were analyzed by Mann–Whitney test. **p < 0.001 versus each condition. These data are representative of three independent experiments (original magnification, × 200; scale bar = 10 μm)

MSCs stimulated by LPS induce the recruitment of neutrophils through the secretion of IL-8 and macrophage migration inhibitory factor (MIF) [30]. Indeed, we demonstrated that after LPS administration, more neutrophils were notably recruited to the liver in the hUCB-MSC-treated condition than in the hUCB-MSC-only condition (Fig. 3a, lower panels; Additional file 6: Videos S3 and Additional file 7: S4). It has been reported that many i.v. injected hMSCs remain mostly intact despite repeated contact with host neutrophils. However, some hMSCs are cleaved and subsequently phagocytized by surrounding GFP^+^ granulocytes in the LPS-stimulated LysM-GFP^+/−^ mice [31]. In the hUCB-MSC-treated condition, we analyzed dynamic interactions between neutrophils and hUCB-MSCs in the inflamed liver of LysM-GFP^+/−^ mice. We observed that neutrophils migrated toward hUCB-MSCs when stimulated with LPS (Fig. 4a; Additional file 8: Video S5). Some neutrophils gathering toward hUCB-MSCs attempted to phagocytize hUCB-MSCs (Fig. 4b; Additional file 9: Video S6 and Additional file 10: S7). Subsequently, neutrophils that engulfed some hUCB-MSCs migrated from the original site to another site (Fig. 4c; Additional file 11: Video S8). After cleavage by neutrophils, the cellular debris of hUCB-MSCs were observed as particles engulfed by neutrophils. These neutrophils showed a significant increase in the contact frequency with other adjacent neutrophils (Fig. 4d; Additional file 12: Video S9). According to quantitative analysis, the relative contact frequency among the neutrophils was markedly increased 6 h after LPS administration in the hUCB-MSC-treated condition compared to that in the hUCB-MSC-only condition (Fig. 4e). This unique phenomenon is the result of neutrophil stimulation by activated MSCs through microbial triggering [32]. Interestingly, neutrophils can survive much longer while interacting with other cells or under pathological conditions such as sepsis [12]. Indeed, more neutrophils gathered around hUCB-MSCs that were not entirely engulfed by numerous neutrophils for several hours during imaging (Fig. 4f; Additional file 13: Video S10 and Additional file 14: S11). In this experiment, we defined the early phase as 4 min and the late phase as 68 min during imaging (Fig. 4f; Additional file 13: Video S10). Quantitative analysis showed that the number of neutrophils while swarming was significantly increased in the late phase compared to that in the early phase (Fig. 4g). This swarm-like migration pattern of neutrophils is called “neutrophil swarming” [33]. This observation could suggest that neutrophils induce swarming behavior through continuous contact or paracrine effects among neutrophils. Several studies have reported that neutrophils are selectively recruited by LPS-stimulated MSCs through paracrine effects. Thus, LPS-triggered MSCs selectively attract neutrophils, thereby strengthening the function and survival of neutrophils [30, 34]. These data indicate that hUCB-MSCs have beneficial effects on LPS-induced sepsis through various biological behaviors in association with neutrophils.

### HSCs enhance the therapeutic effects on LPS-induced sepsis by hUCB-MSCs

**Fig. 5:**
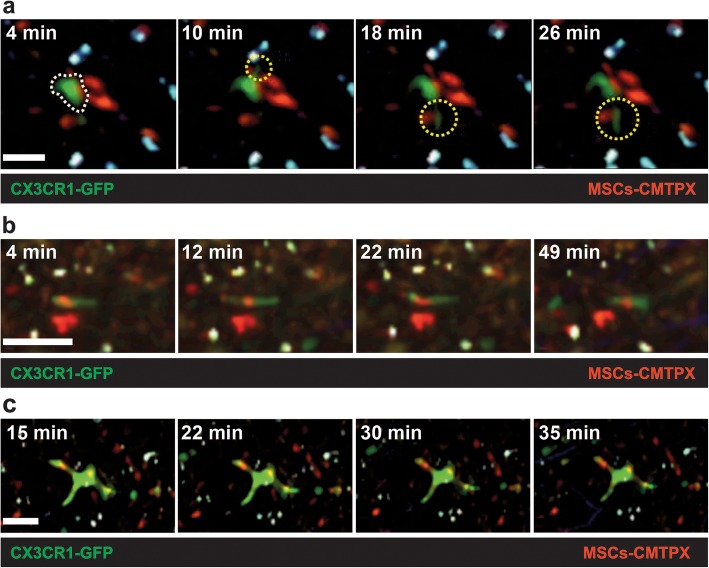
Vigorous interactions between HSCs and hUCB-MSCs in the liver of CX3CR1-GFP^+/−^ mice. **a** Representative time-lapse images showing that HSCs formed extended and retracted dendrites toward hUCB-MSCs. Imaging times (white dotted line: morphology of HSC; yellow dotted line: extended dendrites of HSC; scale bar = 20 μm; Additional file 15: Video S12) are represented. **b** Representative time-lapse images showing that migratory HSCs engulfed hUCB-MSCs under physiological conditions. Imaging times (scale bar = 20 μm; Additional file 16: Video S13) are represented. **c** Representative time-lapse images showing the immobile HSCs that engulfed hUCB-MSCs under pathological conditions (including LPS stimulation). Red: CMTPX-labeled hUCB-MSCs (exogenous signal); green: resident CX3CR1+ HSCs (endogenous signal). Imaging times (scale bar = 20 μm; Additional file 17: Video S14) are represented. Data are representative of three independent experiments (original magnification, × 200)

Hepatic stellate cells (HSCs) are located in the subendothelial space called the space of Disse, between hepatocytes and liver sinusoidal endothelial cells (LSECs) [35]. HSCs have a stellate phenotype characterized by several dendritic processes [35]. In a steady state, HSCs store vitamin A in cytoplasmic lipid droplets. However, in pathological conditions such as hepatic fibrosis and cirrhosis, HSCs lose lipid and vitamin A storage ability and are transformed into myofibroblasts. This change into collagen-producing cells leads to portal hypertension [36], which provides evidence that HSCs perform an essential role in liver immune functions. Hence, it is crucial to investigate interactions between HSCs and hUCB-MSCs in liver immunobiology. To examine any correlation between HSCs and hUCB-MSCs in the liver during LPS-induced sepsis, we performed two-photon intravital imaging 30 h after hUCB-MSC administration in CX3CR1-GFP^+/−^ mice with LPS treatment for 6 h. Recent studies have shown monocyte-derived CX3CR1^+^ LCMs (liver capsular macrophages) [37] and resident CX3CR1^+^ HSCs [4] in the inflamed liver of CX3CR1-GFP^+/−^ mice. Our preliminary experiments demonstrated that LCMs occupied the hepatic capsule with mostly second harmonic generation (SHG) in the inflamed liver of CX3CR1-GFP^+/−^ mice. Second harmonic generation (SHG) has been used to see fibrillar collagen structures in connective tissues in vivo experiments using two-photon intravital imaging [38]. On the other hand, HSCs were located in the space of Disse in the inflamed liver of CX3CR1-GFP^+/−^ mice (Additional file 3: Figure S3a; Additional file 20: Video S17 and Additional file 21: S18). However, we did not detect any notable differences in biological behaviors between LCMs and hUCB-MSCs in the liver of CX3CR1-GFP^+/−^ mice under both physiological and pathological conditions (data not shown). This observation indicates that hUCB-MSC efficacy in mitigating sepsis might be due to interactions with other immune cells rather than monocyte-derived macrophages in the liver. HSCs have been reported to undergo direct contact with disease-associated lymphocytes [39]. HSCs represent 5–8% of hepatic cells in a steady state [40]. Under pathological conditions such as tissue injury and inflammation, HSCs transdifferentiate into a myofibroblast-like phenotype [41–43]. Based on two-photon intravital imaging of the liver of CX3CR1-GFP^+/−^ mice in a time-lapse manner, a few HSCs were mainly observed in the subendothelial space in the absence of LPS treatment (Additional file 3: Figure S3b, upper left panel; Additional file 22: Video S19). Indeed, LPS-induced sepsis certainly promoted the transdifferentiation of HSCs into myofibroblast-like cells (Additional file 3: Figure S3b, upper right panel, Additional file 23: Video S20). However, the number of HSCs was not significantly different in the LPS-treated condition compared to that in control. This indicates that LPS-induced sepsis promotes HSC transdifferentiation into myofibroblast-like cells. Next, we found almost no difference for transdifferentiation capacity of HSCs in the hUCB-MSC-treated condition compared to that in the LPS-treated condition (Additional file 3: Figure S3b, lower left panels; Additional file 23: Video S20 and Additional file 24: S21). According to a previous study, HSCs have been reported to contact a large number of hepatocytes, adjacent stellate cells, endothelial cells, and nerve endings using their cytoplasmic processes [44]. Actually, in the hUCB-MSC-treated condition, we observed vigorous interactions between HSCs and hUCB-MSCs in the liver of CX3CR1-GFP^+/−^ mice. During imaging, we observed direct contact between HSCs and hUCB-MSCs through repeated dendrite movements such as extension and retraction in HSCs (Fig. 5a; Additional file 15: Video S12). We also observed distinct differences in HSC motility between physiological and pathological conditions. First, HSCs engulfing hUCB-MSCs were highly motile and actively crawled through the interstitial tissue of the liver under physiological conditions (Fig. 5b; Additional file 16: Video S13). Second, completely immobile HSCs engulfed hUCB-MSCs under pathological conditions induced by LPS stimulation (Fig. 5c; Additional file 17: Video S14). Interestingly, we observed that the transdifferentiation capacity of HSCs notably decreased in the hUCB-MSC-only condition compared to that in LPS-only and hUCB-MSC-treated conditions (Additional file 3: Figure S3b, lower right panel; Additional file 25: Video S22). This finding implies that HSCs do not recognize hUCB-MSCs as foreign in the mouse body and consequently do not cause graft rejection. In addition, under physiological conditions, there was no significant difference in the motility of MSC debris-containing (Fig. 5b; Additional file 16: Video S13) and debris-free HSCs (Additional file 3: Figure S3b, upper left panel; Additional file 22: Video S19). These observations suggest that the factor of affecting the transdifferentiation and motility of HSCs is not the hUCB-MSCs but the inflammatory environment such as sepsis. According to recent researches, HSCs do not function as APCs under physiological conditions, but this situation has changed in the inflamed liver. Exposure of HSCs to the inflammatory environment changes the morphology of HSCs, strengthening their capacity to present antigen to lymphocytes [45–47]. As a result, HSCs are crucial players to resolve the pathological conditions such as hepatic fibrosis, cirrhosis, and bacterial infection in the liver. These data indicate that HSCs may enhance the therapeutic effects on LPS-induced sepsis by hUCB-MSCs. Therefore, we plan to study the role of HSCs in diverse inflammatory diseases of the liver in future researches.

## Discussion

Sepsis is characterized by an overwhelming systemic inflammatory response due to the PAMPs from invading microorganisms or injured host tissue [48]. PAMPs bind to pattern recognition receptors (PRRs) expressed on innate immune cells, which leads to a hyper-inflammatory response [49]. The activation of PRRs promotes the production of diverse pro-inflammatory molecules such as IL-1β, IL-2, IL-6, IL-8, TNF-α, and IFN-γ and anti-inflammatory cytokines such as IL-10 [34]. This exaggerative production of pro- and anti-inflammatory cytokines and chemokines lead to the “cytokine storm” phase causing severe inflammatory responses [50]. MSC treatment against excessive inflammatory responses has been associated with alleviated systemic pro-inflammatory cytokines, attenuated organ injury, and improved survival in both microbial and polymicrobial sepsis models [51, 52]. We confirmed similar efficacy for hUCB-MSC treatment in microbial sepsis-induced morbidity and mortality. Interestingly, a number of studies have shown that the beneficial effects of MSCs on LPS- or cecal ligation and puncture (CLP)-induced sepsis are linked to an increase in the anti-inflammatory cytokine IL-10 [9, 53]. We also observed that systemic IL-10 levels were markedly elevated by hUCB-MSC treatment. Hereby, in this study, we demonstrated that hUCB-derived MSCs modulate neutrophil migration and improve survival in a microbial sepsis model induced by LPS treatment. This alleviation may result from the immunomodulatory properties of hUCB-MSCs. Several studies have shown that neutrophils can acquire the capacity to function as APCs under inflammatory conditions or during associations with other cells [12, 54]. Thus, these observations suggest that neutrophils mediate innate and adaptive immunity by increasing contact frequency with other cells. Several studies have shown that MSCs have immunosuppressive and immunomodulatory functions. This MSC-mediated immune suppression and modulation has been reported to enable immune evasion [55, 56]. The enhanced immunosuppressive properties of MSCs allow them to mitigate inflammation and delay or avoid host immune rejection by inhibiting T cell responses and suppressing APC maturation [57, 58]. This observation could suggest that hUCB-MSCs have host innate immune evasion or resistance capacity.

HSCs are astral cells that reside in the space of Disse. These HSCs constitute as little as 5–8% of whole liver cells [59]. They also perform a critical role as immune sentinels of the liver [35] and activated HSCs induce hepatic inflammation through expressing MCP-1 [60] and IL-6 [61]. Activated HSCs secrete numerous pro-inflammatory cytokines and chemokines with chemoattractant activity toward leukocytes [47, 60, 62]. Activated HSCs also have been reported to highly express HLA family molecules (predominantly HLA class II) and CD40 for antigen presentation [63]. According to previous studies, HSCs function as nonprofessional APCs such as LSECs [63]. Whereas professional APCs have immune properties under physiological conditions, nonprofessional APCs mainly obtain such characteristics under pathological conditions. Nonprofessional APCs support the immune system in inflamed tissues with increase pro-inflammatory cytokine and chemokine production. For example, HSCs can phagocytose macromolecules and bacteria [63]. Indeed, we observed that HSCs directly contact hUCB-MSCs through repeated dendrite movements such as extension and retraction in HSCs. Previous studies have shown that skin dendritic cells (termed Langerhans) and microglial cells function as sentinels against invading microorganisms showing similar dendrite behaviors, termed “dSEARCH (dendrite surveillance extension and retraction cycling habitude)” [37]. In this study, HSCs did not recognize hUCB-MSCs as foreign in the mouse body and consequently did not cause graft rejection. These findings indicate that HSCs may enhance the therapeutic effects on LPS-induced sepsis by hUCB-MSCs.

Over the past few years, clinical trials with MSC therapy have been reported in diverse inflammatory diseases such as various autoimmune diseases [64, 65], graft-versus-host disease (GvHD) [66], ulcerative colitis [34], acute respiratory distress syndrome (ARDS) [67], and septic shock [50]. Despite numerous studies on their efficacy, the mechanism underlying the therapeutic effects of MSCs in diverse inflammatory diseases has not yet been fully elucidated. However, an understanding of the importance of the immunomodulatory properties of MSCs for sepsis treatment is becoming elucidated [55, 68]. According to previous studies, these immunomodulatory properties also enable immune evasion from the host immune system [55, 56]. Similar to these studies, the immunomodulatory properties of hUCB-MSCs are required for their proper application to inflammatory responses. Particularly, the persistence of MSC effects is correlated with the rate of immune detection [55]. The rates of MSC immune detection and elimination are governed by a balance between the relative expression of immunogenic and immunomodulatory factors in MSCs. In other words, a decrease in immunogenic factors [18] leads to slower immune detection [55]. Together, the immunomodulatory properties of MSCs enable evasion from immune rejection responses. In fact, graft rejection is a common phenomenon when human cells are injected into a mouse. However, in our experiments with hUCB-MSCs, we hardly detected such graft rejection. In clinical trials of MSCs for sepsis treatment, the rejection of transplanted cells occurs less often and later than that of other transplanted tissues. For this reason, cellular therapy using the immunomodulatory properties of MSCs for sepsis treatment could be favorable.

## Conclusion

This study is significant as it shows biological behaviors of neutrophils and hUCB-MSCs in live murine sepsis model in a real-time manner. Further, our research would contribute to future studies on the mechanism underlying neutrophil and MSC interactions with respect to the treatment of sepsis.

## Supplementary information

**Additional file 1: Figure S1.** Characterization of hUCB-MSC. a. In vitro culture, hUCB-MSC morphology. Low density and high density hUCB-MSCs (original magnification × 100; scale bar = 200 μm). b. hUCB-MSCs were characterized by flow cytometry and immunofluorescent staining for CD73, CD90, and α-SMA.

**Additional file 2: Figure S2.** Neutrophils and hUCB-MSCs interactions in the inflamed liver between the two different LPS doses (0.5 mg/kg and 25 mg/kg) of LysM-GFP^+/−^ mice. a. Representative images for each dose; red: CMTPX-labeled hUCB-MSCs (exogenous signal); green: neutrophils (endogenous signal). The two different LPS doses (0.5 mg/kg and 25 mg/kg) of hUCB-MSCs-treated condition in 24 h post-LPS injection. The 0.5 mg/kg of LPS (Video S15) and 25 mg/kg of LPS (Video S16). These data are representative of three independent experiments (original magnification, × 200; scale bar = 30 μm). b. The graph shows the number of neutrophils per FOV (mm^3^) for each dose (mpk = mg/kg; n.s. = not significant). Quantitative results indicate the average values ± SD of at least three independent experiments. The results were analyzed by a Mann-Whitney test.

**Additional file 3: Figure S3.** Liver capsular macrophages (LCMs) and Hepatic stellate cells (HSCs) in the liver of CX3CR1-GFP^+/−^ mice. a. Representative images for LCMs and HSCs (Video S17 and S18); blue: second harmonic generation (SHG, endogenous signal); green: LCMs (left panel) and HSCs (right panel) (endogenous signal). b. Representative images for each condition; red: CMTPX-labeled hUCB-MSCs (exogenous signal); blue: second harmonic generation (SHG, endogenous signal); green: HSCs (endogenous signal). Conditions included control (Video S19), LPS (LPS-only-treated; Video S20), hUCB-MSCs + LPS (hUCB-MSCs-treated; Video S21), and hUCB-MSCs (hUCB-MSCs only treated; Video S22). The “guiding arrows” represent HSCs in Figure S3a and b. These data are representative of three independent experiments (original magnification, × 200; scale bar = 40 μm).

**Additional file 4: Video S1.** Two-photon intravital image of control condition in the liver of LysM-GFP^+/−^ mice.

**Additional file 5: Video S2.** Two-photon intravital image of LPS-only treated condition in the liver of LysM-GFP^+/−^ mice.

**Additional file 6: Video S3.** Two-photon intravital image of hUCB-MSCs + LPS condition in the liver of LysM-GFP^+/−^ mice.

**Additional file 7: Video S4.** Two-photon intravital image of hUCB-MSCs only condition in the liver of LysM-GFP^+/−^ mice.

**Additional file 8: Video S5.** Two-photon intravital image showed that neutrophils migrated toward hUCB-MSCs after LPS stimulation.

**Additional file 9: Video S6.** Two-photon intravital image showed that neutrophils gathered toward hUCB-MSCs attempted to phagocytize hUCB-MSCs.

**Additional file 10: Video S7.** Three-dimensional image of video S6 at a timepoint.

**Additional file 11: Video S8.** Two-photon intravital image showed that neutrophil engulfed hUCB-MSCs migrated from the original site to another site.

**Additional file 12: Video S9.** Two-photon intravital image showed that neutrophils having hUCB-MSCs showed frequent contacts with adjacent other neutrophils.

**Additional file 13: Video S10.** Two-photon intravital image of swarm-like migration pattern of neutrophils.

**Additional file 14: Video S11.** Three-dimensional image of video S10 at a timepoint.

**Additional file 15: Video S12.** Two-photon intravital image showed that HSCs formed repeated dendrite movement toward hUCB-MSCs (Repeated dendrites movement).

**Additional file 16: Video S13.** Two-photon intravital image showed that migratory HSCs engulfed hUCB-MSCs under physiological conditions.

**Additional file 17: Video S14.** Two-photon intravital image showed that completely immobile HSCs engulfed hUCB-MSCs under LPS stimulation.

**Additional file 18: Video S15.** Two-photon intravital image of hUCB-MSCs + LPS (0.5 mg/kg) condition in the liver of LysM-GFP^+/−^ mice.

**Additional file 19: Video S16.** Two-photon intravital image of hUCB-MSCs + LPS (25 mg/kg) condition in the liver of LysM-GFP^+/−^ mice.

**Additional file 20: Video S17.** Two-photon intravital image of LCMs in the liver of CX3CR1-GFP^+/−^ mice.

**Additional file 21: Video S18.** Two-photon intravital image of HSCs in the liver of CX3CR1-GFP^+/−^ mice.

**Additional file 22: Video S19.** Two-photon intravital image of control condition in the liver of CX3CR1-GFP^+/−^ mice.

**Additional file 23: Video S20.** Two-photon intravital image of LPS-only treated condition in the liver of CX3CR1-GFP^+/−^ mice.

**Additional file 24: Video S21.** Two-photon intravital image of hUCB-MSCs + LPS condition in the liver of CX3CR1-GFP^+/−^ mice.

**Additional file 25: Video S22.** Two-photon intravital image of hUCB-MSCs only condition in the liver of CX3CR1-GFP^+/−^ mice.

## Abbreviations

hUCB-MSCs: Human umbilical cord blood-derived mesenchymal stem cells
LPS: Lipopolysaccharide
HSCs: Hepatic stellate cells
APCs: Antigen-presenting cells
DAMPs: Damage-associated molecular patterns
PAMPs: Pathogen-associated molecular patterns
PRRs: Pattern recognition receptors
α-SMA: Alpha-smooth muscle actin
mpk: mg/kg
SHG: Second harmonic generation
