# Supplementary figures and images for: In vivo monitoring of dynamic interaction between neutrophil and human umbilical cord blood-derived mesenchymal stem cell in mouse liver during sepsis
Source: Stem Cell Res Ther. 2020 Feb 3;11:44. doi: 10.1186/s13287-020-1559-4 (PMC6998265; doi:10.1186/s13287-020-1559-4)

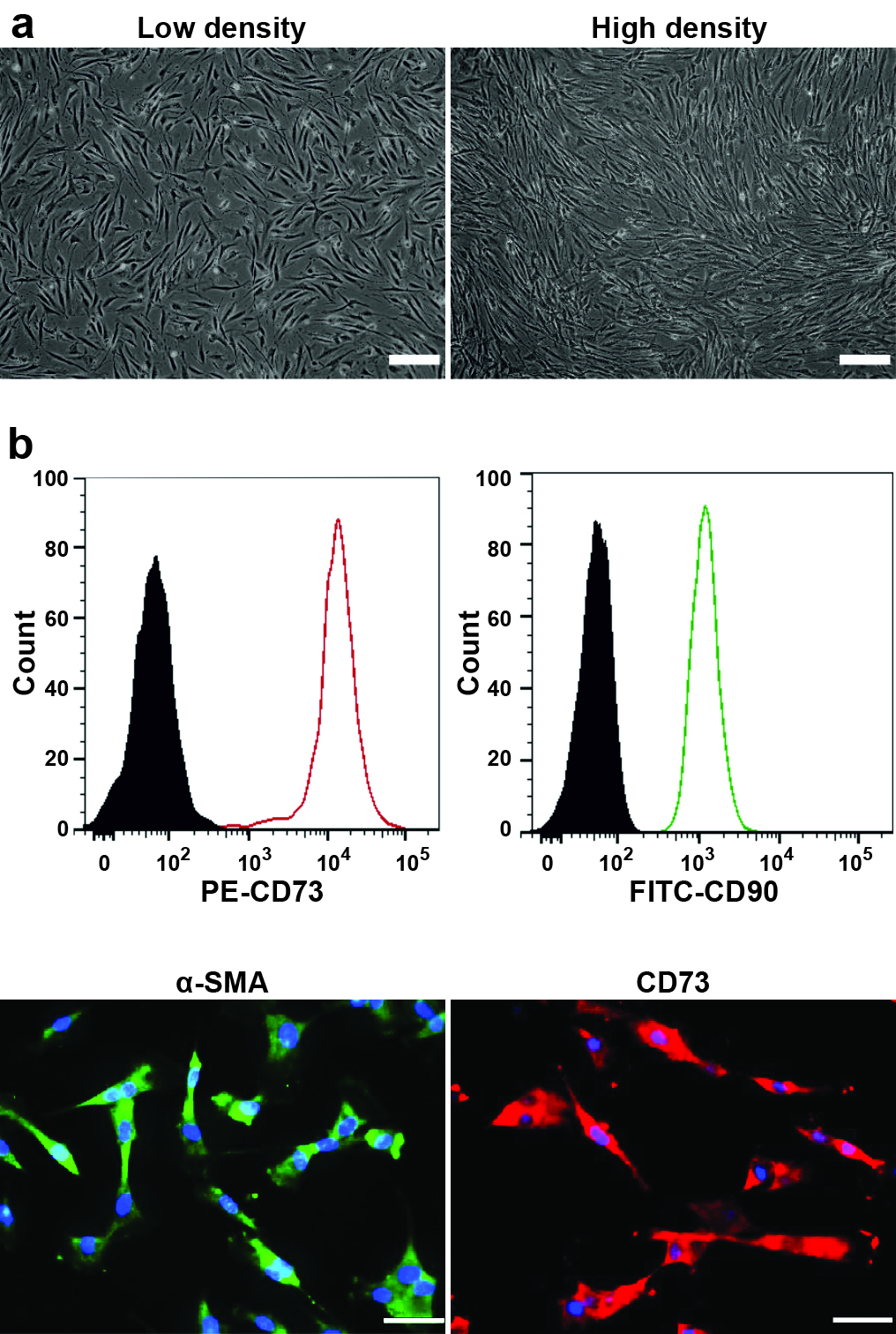

Supplement: Supplementary file 1 — Additional file 1: Figure S1. Characterization of hUCB-MSC. a. In vitro culture, hUCB-MSC morphology. Low density and high density hUCB-MSCs (original magnification × 100; scale bar = 200 μm). b. hUCB-MSCs were characterized by flow cytometry and immunofluorescent staining for CD73, CD90, and α-SMA. [file 13287_2020_1559_MOESM1_ESM.jpg]

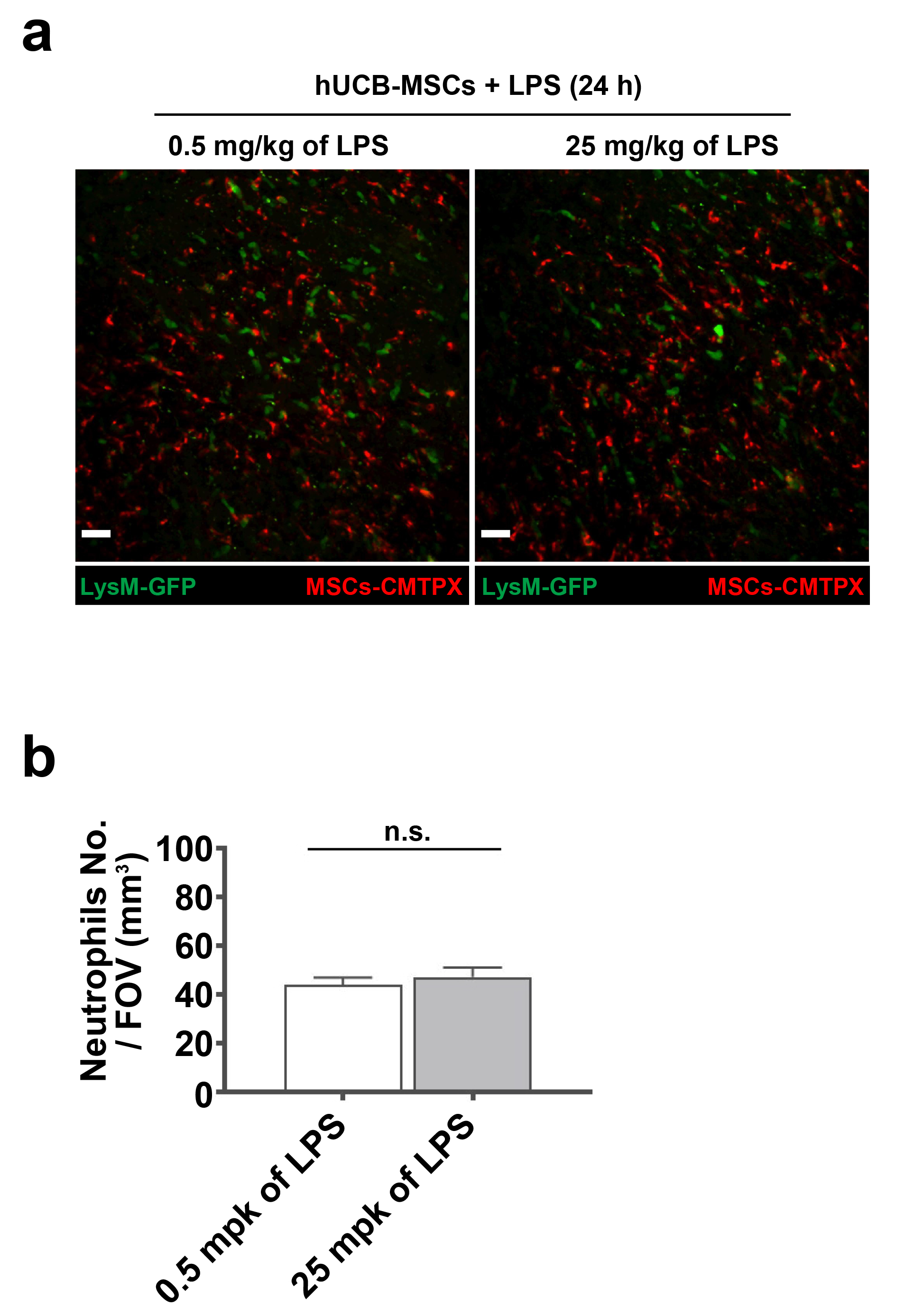

Supplement: Supplementary file 2 — Additional file 2: Figure S2. Neutrophils and hUCB-MSCs interactions in the inflamed liver between the two different LPS doses (0.5 mg/kg and 25 mg/kg) of LysM-GFP+/− mice. a. Representative images for each dose; red: CMTPX-labeled hUCB-MSCs (exogenous signal); green: neutrophils (endogenous signal). The two different LPS doses (0.5 mg/kg and 25 mg/kg) of hUCB-MSCs-treated condition in 24 h post-LPS injection. The 0.5 mg/kg of LPS (Video S15) and 25 mg/kg of LPS (Video S16). These data are representative of three independent experiments (original magnification, × 200; scale bar = 30 μm). b. The graph shows the number of neutrophils per FOV (mm3) for each dose (mpk = mg/kg; n.s. = not significant). Quantitative results indicate the average values ± SD of at least three independent experiments. The results were analyzed by a Mann-Whitney test. [file 13287_2020_1559_MOESM2_ESM.jpg]

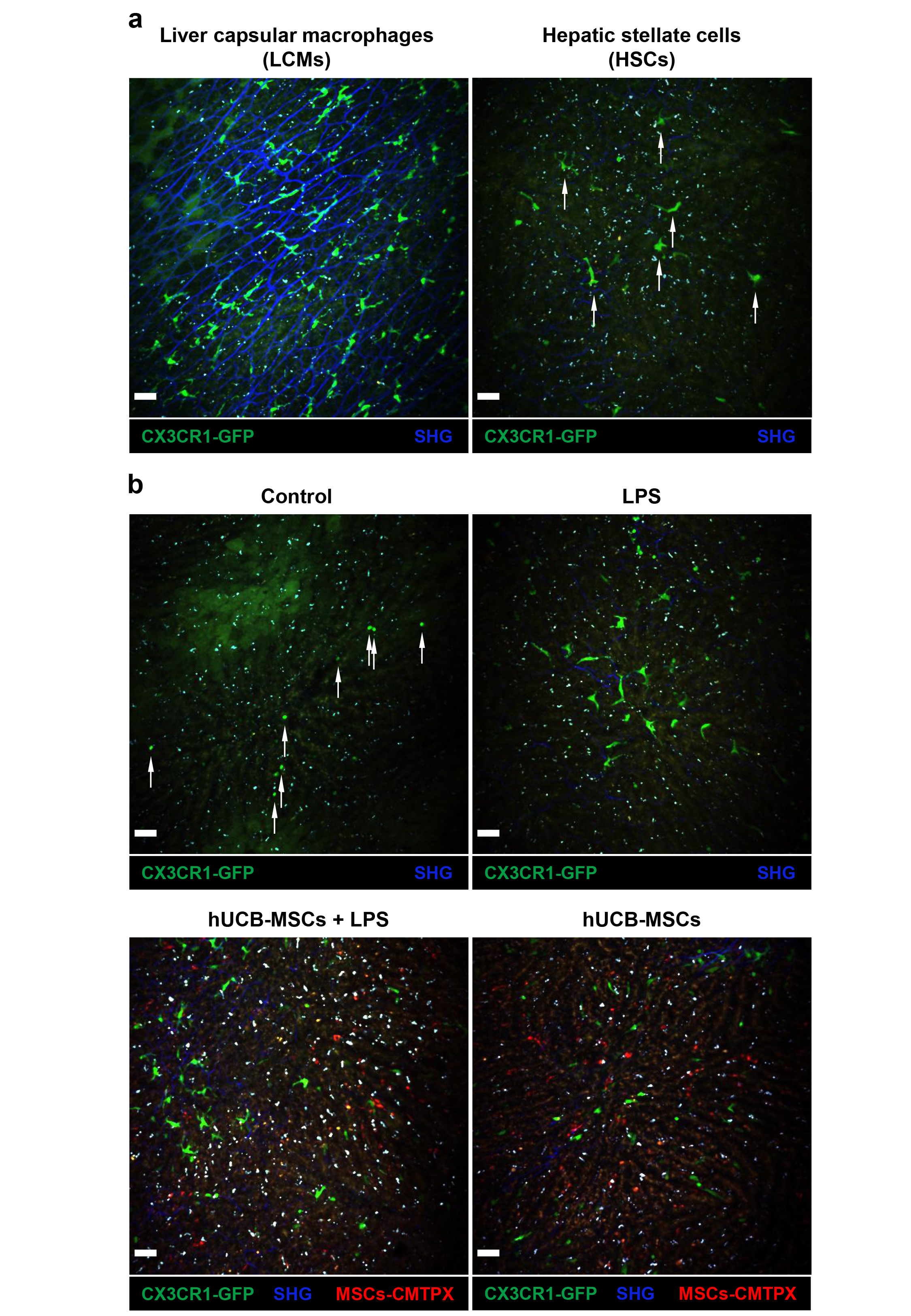

Supplement: Supplementary file 3 — Additional file 3: Figure S3. Liver capsular macrophages (LCMs) and Hepatic stellate cells (HSCs) in the liver of CX3CR1-GFP+/− mice. a. Representative images for LCMs and HSCs (Video S17 and S18); blue: second harmonic generation (SHG, endogenous signal); green: LCMs (left panel) and HSCs (right panel) (endogenous signal). b. Representative images for each condition; red: CMTPX-labeled hUCB-MSCs (exogenous signal); blue: second harmonic generation (SHG, endogenous signal); green: HSCs (endogenous signal). Conditions included control (Video S19), LPS (LPS-only-treated; Video S20), hUCB-MSCs + LPS (hUCB-MSCs-treated; Video S21), and hUCB-MSCs (hUCB-MSCs only treated; Video S22). The “guiding arrows” represent HSCs in Figure S3a and b. These data are representative of three independent experiments (original magnification, × 200; scale bar = 40 μm). [file 13287_2020_1559_MOESM3_ESM.jpg]
